# Targeting the Highly Invasive Malaria Vector Anopheles stephensi using Yeast RNAi Pesticides

**DOI:** 10.21203/rs.3.rs-8982626/v1

**Published:** 2026-03-02

**Authors:** Teresia M. Njoroge, Majidah Hamid-Adiamoh, Keshava Mysore, Akilah T. M. Stewart, Longhua Sun, Darlene D. Akaiso, Molly Duman-Scheel

**Affiliations:** Indiana University School of Medicine; Indiana University School of Medicine; Indiana University School of Medicine; Indiana University School of Medicine; Indiana University School of Medicine; University of Notre Dame; Indiana University School of Medicine

**Keywords:** RNAi, yeast, insecticide, larvicide, ATSB, Anopheles stephensi, sex-separation, malaria

## Abstract

**Background:**

Apart from widespread resistance of malaria mosquitoes to insecticides, *Plasmodium* parasite resistance to frontline anti-malaria drugs, and challenges in malaria diagnosis, the World Health Organization (WHO) has described the highly invasive *Anopheles stephensi* as a major threat to malaria control. New classes of insecticides are vitally needed for integrated control of the dangerous malaria vector that continues to spread across African countries. Yeast RNAi insecticides are promising novel pesticides that could prove effective for integrated responses to *A. stephensi*. Here we explore the use of RNAi yeast pesticides for control of this invasive malaria vector.

**Methods:**

Sh.463, a modified *Saccharomyces cerevisiae* baker’s yeast RNAi pesticide corresponding to the *A. stephensi Shaker* gene, was evaluated in *A. stephensi*. A scalable attractive targeted sugar bait (ATSB)-based system for delivery of *Sh* interfering RNA pesticides (IRPs) to adult *A. stephensi* under insectary conditions is examined, and a yeast-based system for delivery of *Sh* IRP to larvae is developed and evaluated. Additionally, female-specific yeast RNAi-larvicides targeting putative *A. stephensi* female-specific genes for male mosquito sorting are also developed and evaluated in laboratory assays.

**Results:**

We demonstrate that the treatment of *A. stephensi* larvae and adults with Sh.463–56.10R yeast silences the mosquito *Shaker* gene, resulting in high levels of mortality in laboratory studies. Additionally, our siRNA screens of putative female-specific genes in *A. stephensi* using female-specific RNAi yeast larvicides resulted in significant female mortality in cup bioassays leading to significantly higher male: female ratios in the resulting offspring.

**Conclusion:**

The results of these studies suggest that an RNAi pesticide targeting mosquito *Shaker* genes may represent a novel biorational intervention that can be used in integrated *A. stephensi* control programs while also targeting other species of disease vector mosquitoes. The potential of *A. stephensi* female-specific RNAi yeast larvicides in male mosquito sorting is also described.

## INTRODUCTION

Malaria, a serious and sometimes fatal illness, is a huge economic and public health burden globally, with an estimated 263 million cases and approximately 600,000 malaria deaths annually [1]. Although malaria affects at least 80 countries worldwide, the African region carries a proportionately high share of the global malaria burden, with 94% of malaria cases occurring there, and children under age five accounting for about 76% of all malaria deaths [1]. Malaria is transmitted by the bite of infective female *Anopheles* mosquitoes that vector protozoan parasites of the genus *Plasmodium*. Although about 60 species of *Anopheles* are known competent vectors of the *Plasmodium* parasites, under natural conditions only 30 are of major significance [2]. Historically, the *Anopheles gambiae complex* and *Anopheles funestus* species, occur predominantly in Africa and are responsible for malaria transmission in the highly malaria endemic region [3–5]. In Latin America, *A. darlingi* and *A. marajoara* are among the primary vectors of malaria [6–9]. In the Asian-pacific region that includes South Asia and the Middle East, several dozen malaria vector species exist, both zoophilic and exophilic [10], and *A. stephensi* has been recognized as an important vector of malaria in urban areas across the region [10–12].

*A. stephensi* is domicile to the Asian continent where it has been reported as one of the major drivers of malaria in the region. The malaria mosquito has also colonized at least eight countries in sub-Saharan Africa (SSA) since 2012 and is now spreading at an alarming rate [13]. *A. stephensi* is a competent vector of *Plasmodium falciparum* and *Plasmodium vivax*, which are the primary malaria parasites in SSA, and has been implicated in malaria epidemics in Djibouti [14],[15] and Ethiopia [16]. Unlike other malaria vectors, *A. stephensi* exhibits high ecological plasticity and is considered both an urban and peri-urban adapted malaria vector, which breeds in artificial water containers such as overhead tanks, cisterns, ditches and canals [17], [18]. Although malaria in Africa has been predominantly a rural disease, it is expected that transmission will rise in the rapidly growing urban areas where *A. stephensi* populations establish, calling for an urgent need for integrated responses to control the invasive malaria vector. In 2023, the WHO[19] released a threat notice highlighting the spread of *A. stephensi* in the Horn of Africa and advised public health authorities to heighten vector surveillance efforts. New tools are vitally needed to combat the dangerous malaria vector.

Vector-targeted interventions that mostly involve use of chemical insecticides have proven effective in preventing malaria transmission [20], but this vector control approach is currently faced with challenges that warrant the discovery of new tools for integrated vector management. Just like other disease vector mosquitoes, the *A. stephensi* strains that were detected in Africa are largely resistant to commonly used insecticides and prefer to bite people outdoors, making bed nets and indoor residual spraying, the mainstays of malaria vector control, less effective for combatting the invasive malaria vector [21]. Furthermore, there are serious concerns regarding public health and the environmental safety of chemical-based pesticides [22, 23] and hence new classes of pesticides that are effective and environmentally safe are vitally needed. RNA interference (RNAi)-based pesticides are promising novel insect pest management tools with the potential to be both safe and effective. These pesticides employ RNAi, an innate regulatory mechanism occurring in eukaryotes, that enables targeted gene silencing in many organisms, including mosquitoes. For the past 25 years, RNAi technology has advanced as a useful research tool with the potential to translate innovative bench research into practical pest control strategies [24, 25]. The objective of this research was to evaluate the potential for RNAi yeast-based technologies to facilitate the control of *A. stephensi*.

In recent years, we have pursued larvicide[26] and adulticide[27] screens for RNAi insecticides. In the present study, we evaluate a novel mosquito control technology in *A. stephensi*, in which we investigate the insecticidal activity of Sh.463, an RNAi pesticide consisting of a short hairpin RNA (shRNA) expressed in yeast that targets the mosquito *Shaker* (*Sh*) gene [28]. The insecticidal yeast is propagated, heat killed and lyophilized then evaluated in larvae and adult *A. stephensi*. Mosquito larvae consume the yeast readily as part of their diet, and for the adults, the insecticidal yeast is mixed with sugar and presented to sugar seeking adults as an ATSB. Following mosquito consumption, the RNAi yeast was designed to silence the *Sh* gene, which is expressed widely in Dipteran nervous systems. The neuron-specific gene encodes an evolutionarily conserved subunit of a voltage-gated potassium channel responsible for membrane repolarization and release of neurotransmitters in the brain [29]. Sh.463 recognizes a conserved *Sh* target site found in *Aedes, Culex*, and *Anopheles* mosquitoes, including *A. stephensi* [28], but not in non-target organisms. Based on previous analyses of the insecticidal activity of Sh.463 yeast, as well as Sh.463–56.10R, a robust strain with enhanced Sh.463 shRNA production [30], in larvae and adult *A. gambiae, Aedes*, and *Culex* mosquitoes [28], it was hypothesized that Sh.463–56.10R would silence the *A. stephensi Sh* gene, resulting in death of both larvae and adults.

In addition to identifying genes to be targeted for killing larvae and adults, we also performed screens in *Aedes aegypti* that identified female-specific RNAi larvicides [31, 32], some of which target female larval lethal genes such as *MtnB*[31] and *GGT*[32] that are conserved in different mosquito species, to facilitate male sex separation. It is hypothesized that targeting the *A. stephensi* orthologs of these or other female-specific larval lethal genes might enable male sex separation. This would facilitate deployment of the sterile insect technique (SIT) or other population control methods. Although other methods for male sex sorting of mosquitoes have been developed [33–35], the use of female-specific yeast methodology requires only the addition of dried RNAi yeast to the larval diet during rearing, which yields female deaths and up to 5 male:1 female sex ratios in adults. The adult males produced by this technology have shown no loss of fitness in laboratory experiments [31]. RNAi yeast-based diets that facilitate scaled rearing and separation of *Aedes* and *Culex* males have been developed [31], [32, 35], suggesting that this technology can be adapted for use in *A. stephensi*.

In summary, the goals of this study are to assess whether RNAi yeast technology can be applied for larviciding, adulticiding (through ATSBs), and male sex separation in the malaria vector mosquito *A. stephensi*.

## METHODS

### 1. Mosquito rearing

The *A. stephen*s*i* strain used in this investigation was obtained from BEI Resources (NIAID, NIH: *A. stephensi*, Strain STE2, Eggs, MRA-128, contributed by Mark Q. Benedict). This mosquito strain was reared as described [36], except that the adult females were blood fed using a Hemotek artificial membrane feeding system (Hemotek Limited, Blackburn, UK) to deliver sheep blood that was purchased from HemoStat Laboratories, Dixon, CA. The insectary used for mosquito rearing and insecticidal assays was maintained at 26°C, ~ 80% relative humidity, with a 12 hr dark/12 hr light cycle that included 1hr crepuscular periods at the beginning and end of each cycle.

### 2. Yeast preparation

The Sh.463–56.10R yeast strain previously described by Brizzee et al. [30] was used in this investigation. This second-generation yeast strain of the original Sh.463 strain [28] bears multiple copies of a high-expression *Sh.463* short hairpin expression cassette, allowing for scaled production of the Sh.463 shRNA, which corresponds to the following target sequence: 5’- AUUUAAAUUAUCUAGGCAUUCGAAA − 3’ in the *A. stephensi Shaker* gene. DMT347.1R, the control RNAi strain used in this investigation, was previously described by Brizzee et al. [30] and recognizes 5’-GAAGAGCACUGAUAGAUGUUAGCGU-3’, a target site that has not been identified in any of the mosquito species [28]. The DMT347.1R yeast strain (hereafter referred to as the control) contains multiple integrations of the shRNA expression cassette [30]. Both the Sh.463–56.10R and control RNAi yeast strains are suitable for scaled RNAi yeast production [30]. Yeast was cultured and prepared for insecticide assays as previously described [37] except that production was scaled in a bioreactor (Chemglass Life Sciences, Vineland, New Jersey, USA) and dried using a freeze-dryer (Labconco, Kansas City, Missouri, USA).

### 3. Larvicide assays

Laboratory larvicide assays were performed according to WHO[38] mosquito larvicidal testing guidelines. A previously described protocol[30] was followed using Sh.463–56.10R insecticidal or control yeast fed to 20 first instar larvae reared in 500 mL plastic cups with 50 mL distilled water according to the WHO protocol. Mortality, pupariation, and adult emergence data were collected. Three biological trials, each with six replicates, were performed for each treatment (total of 360 larvae for each treatment group), and the mortality data were analyzed using SPSS 25 (IBM, Armonk, NY USA) software with the Mann-Whitney U-test.

### 4. Adulticide Studies

The Sh.463–56.10R insecticidal yeast or control yeast was mixed with Westham sugar matrix (Westham LLC, Israel) to make a yeast ATSB insecticide and presented to five-day old adult female *A. stephensi* in the insectary according to a protocol described previously [30]. LC_50_ and LC_90_ concentrations of Sh.463–56.10R yeast ATSB were determined previously [30], and these data were used to select the appropriate dosage for the present investigation. For each feeding treatment of 25 adult female mosquitoes, 20 mg of lyophilized yeast (treatment or control) were placed on a 6 cm × 6 cm piece of a porous Westham membrane (Westham LLC, Israel). A volume of 100 μL of Westham sugar matrix was added to the pre-weighed yeast (20mg) and mixed thoroughly with a toothpick to create a paste on top of the membrane. A second piece of membrane was placed over the yeast and sealed with a heat sealer to create a sachet containing yeast insecticide. The method for preparation of the RNAi yeast sachet is shown in **Fig. S1**. The sachet containing the yeast-ATSB pesticide or control was delivered to mosquitoes in insectary sugar bait trials by placing it at the bottom of a cage containing 25 five-day old adult females that had been starved overnight. Engorged females were assessed daily for behavioral phenotypes, mortality, and morbidity for six days. Three biological trials, each with three replicates, were conducted for each treatment (total of 225 adult mosquitoes for each treatment), and data were analyzed using SPSS 25 (IBM, Armonk, NY USA) software with Kruskal-Wallis test.

### 5. Attractive sugar bait (ASB) choice assays in adult

5. Attractive sugar bait (ASB) choice assays in adult *A. stephensi*

To identify the most attractive sugar bait for effective delivery of RNAi yeast to adult *A. stephensi*, choice assays were conducted in the insectary using a 10% sucrose solution, Westham sugar matrix or soda (Coca-cola^™^) as a sugar source. To achieve this, dual-choice experiments were performed to evaluate mosquito feeding preference which was determined using adult mosquito mortality. The choice experiments were conducted in 3.75 L (30 cm × 30 cm × 30 cm) BugDorm-1 insect rearing cages (MegaView Science Co., Ltd, Taichung, Taiwan) using both treatment yeast and control yeast mixed with either of two sugar sources and simultaneously placed in the cage. An ASB sugar control for each sugar type was also included in separate cages to verify that sugar feeding occurred. All the yeast-sugar bait formulations (yeast-10% sugar, yeast-soda, and yeast-Westham sugar bait) were prepared to a final sugar-yeast concentration of 333.3 μg/μL as described[28] and presented to 25 adult female *A. stephensi* per cage that were allowed to feed on the treatments overnight. For the yeast-10% sugar vs. yeast-soda formulation adult feeding trials, the treatments were administered using MUDUODUO automatic bird drinker cups (Amazon, Seattle Washington) custom-designed as ATSB feeders as described [39]. For the yeast-soda vs. yeast-Westham bait formulation trials, the treatments were presented in the well of an inverted 2 oz transparent polypropylene plastic condiment container with the yeast ATSB covered by a perforated stretched parafilm to facilitate adult mosquito probing. Two yeast treatments bearing different sugar baits were positioned in opposite corners of the experimental cages to ensure equal exposure and unbiased access by the mosquitoes. Each treatment pair was tested in three biological trials, with two replicates each, giving a total of 150 adult *A. stephensi* per treatment group. The location of the treatment and control baits was permutated in these trials. Mortality and morbidity were monitored and recorded for six days. The mortality data were statistically analyzed using SPSS 25 (IBM, Armonk, NY USA) software with the Mann-Whitney U test.

### 6. Confirmation of Shaker gene silencing in larvae and adult

6. Confirmation of Shaker gene silencing in larvae and adult *A. stephensi*

Silencing of the *Shaker* gene target in *A. stephensi* larvae and adults following consumption of Sh.463–56.10R yeast was verified using RT-qPCR, which was performed on mosquitoes that had been treated with control or insecticidal yeast. For the larval assays, total RNA was extracted from pools of 20 third instar larvae that had been treated with Sh.463–56.10R yeast or control yeast. Three biological trials, each with three replicates (total of nine replicates) were conducted for the assay. For the adult assays, total RNA was extracted from individual females (n = 15, generated in three biological replicate trials) that had consumed Sh.463–56.10R yeast ATSB or control yeast ATSB for 96 hours. TRIzol (Invitrogen, Carlsbad, CA) was used to extract total RNA as described in the manufacturer’s instructions; the RNA was then treated with DNase 1 using the DNA-free kit (Invitrogen, Thermo Fisher Scientific, Waltham, MA) according to the manufacturer’s instructions. The High Capacity RNA to cDNA Kit (Applied Biosystems, Foster City, CA) was used to generate cDNA, the amplification of which was performed in a CFX Opus 96 Real-time PCR System (Bio-Rad, Hercules, CA) using the Power SYBR Green PCR Master Mix (Applied Biosystems, Foster City, CA) and the following primers: *Sh*F9 for: 5’ GGCACAAAGATCGAGGAGG 3’ and *Sh*F9 rev: 5’ CCTCTTCTTCGGCGACTAC 3’. The amplification of *A. stephensi ribosomal protein* 7 *(rps7)* was performed with the primers 5′ AGCAGCAGCAGCACTTGATTTG 3′ (forward) and 5′ TAAACGGCTTTCTGCGTCACCC 3′ (reverse)[40] and used for data normalization. The qPCR reactions were performed in three technical replicate wells in each of three separate biological replicate trials. Results from these assays were quantified through standardization of reactions to levels of *rps7* using the ΔΔCt method as described [41]. Treatment and control data were statistically analyzed using Microsoft Excel 365 software with the Student’s t-test.

### 7. Development and evaluation of A. stephensi female-specific larvicides

*A. stephensi* female-specific yeast RNAi pesticides were prepared through characterization of putative *A. stephensi* female-specific larval lethal genes. An siRNA (small interfering RNA) screen was performed using orthologs of *A. aegypti* and *A. gambiae* female-specific larval lethal genes [31], [32],[42] in *A. stephensi*. Custom siRNAs corresponding to the orthologous genes were selected using the Integrated DNA Technologies (IDT) Custom Dicer-Substrate siRNA (DsiRNA) tool [43]. Custom siRNAs, including a control siRNA with no known target in mosquitoes, were purchased from Integrated DNA Technologies (Coralville, IA). In summary, short hairpin RNA expression cassettes corresponding to putative target sites in each gene were designed as described [37]. Custom DNA oligonucleotides corresponding to these sequences were purchased from Invitrogen (Carlsbad, CA) and cloned into pRS426 GPD, a non-integrating bacteria-yeast shuttle vector bearing a *URA3* marker that permits constitutive expression of inserts cloned downstream of a GPD promoter [44]. Following sequencing to confirm the inserted sequences, the plasmids were transformed into the *S. cerevisiae CEN.PK* strain yeast [genotype *MATa/α ura3–52/ura3–52 trp1–289/trp1–289 leu2–3_112/leu2–3_112 his3 Δ1/his3 Δ1 MAL2– 8C/MAL2–8C SUC2/SUC2]* [45], and the transformants were selected by growth on minimal media lacking uracil. The yeast IRPs were then cultured, heat-killed, and dried for larval feedings as described [37].

The *A. stephensi* female-specific IRPs produced were used to perform larvicidal bioassays in accordance with WHO [38] guidelines in the insectary (see above). Adult male and female emergence rates and sexes were evaluated, and data were statistically analyzed with the Chi-square test. The success of population-based mosquito control programs such as SIT depends on male fitness which determines the ability of the released sterile males to compete with the existing wild-type male populations for wild-type females and induce sterility in the target population. To assess *A. stephensi* male fitness following consumption of female-specific yeast larvicides, *A. stephensi* female-specific *doublesex* (*dsx*F) larvicide, which resulted in significant female larval deaths in lab assays was evaluated. *A. stephensi* male fitness following consumption of *dsx*F yeast larvicide was examined through wing length measurements and mating competitiveness assays in the laboratory:

#### a) A. stephensi males wing length assays

To evaluate *A. stephensi* adult males’ fitness following consumption of larval rearing diet containing female-specific yeast larvicides, male wing lengths were estimated compared to mosquitoes grown on the normal rearing diet (food control). *A. stephensi* adult males that had been fed with control diet, *dsx*F.744 or *dsx*F.745 yeasts during the larval stage were generated as described in section (**3**) above. For each of the three treatment groups, three biological trials were conducted, each with 40 males per treatment. Individual adult males’ wings were then mounted on specimen slides in pairs using a clear nail polish. Pictures were taken under a stereo microscope and analyzed using FIJI ImageJ software [46] to determine their wing lengths. The data from the control and two treatment groups were statistically analyzed using ANOVA.

#### b) Competitive mating assays

The mating competitiveness of *A. stephensi* males following consumption of the male sorting dsxF yeast during larval rearing was conducted using Rhodamine B (RhB) (CAS: 81889, ThermoFisher Scientific, Waltham, Massachusetts, USA), a fluorescent dye used as a marker to trace mating. Briefly, virgin adult female *A. stephensi* were crossed with *dsx*F-A.744 or *dsx*F-A.745 yeast-treated and marked (RhB+) and control unmarked males (RhB-) for copulation and their spermathecae dissected and examined for fluorescence to determine the mating partner using a fluorescent microscope following the methodology previously described [47], [48].

Both adult males and females were generated by rearing first instar *A. stephensi* larvae to pupal stage (as described in section 3 above) using the standard mass-rearing (MR) diet, which served as a control (food control), *dsx*F.744 or *dsx*F.745 yeasts. For each treatment, the pupae were placed in individual vials to separate adult males from females. Newly emerged adult mosquitoes from the three treatments were then maintained with 10% sucrose solution with or without RhB for three days in 2 L (16.2 cm × 16.2 cm × 15.5 cm) cages. Specifically, *dsx*F.744 and *dsx*F.745 treated males were marked through sugar feeding using 0.1% w/v RhB-sucrose solution made by dissolving 100mg of RhB powder for 100 mL of 10% w/v sucrose solution, for 3 days. The control males and females reared on their normal diets, hereafter referred to as “wild-type males and females” were each maintained using 10% sucrose solution for three days as well. The mating assays were conducted three days following sugar feeding by crossing 20 RhB marked males (RhB+), 20 unmarked wild-type males (RhB-) with 40 virgin wild-type female mosquitoes for three nights. Three biological replicate trials for each treatment were conducted (a total of 120 females were examined). The females’ spermathecae were then dissected using a dissecting microscope, and their insemination and fluorescence status were determined using a fluorescent microscope. The spermathecae of the inseminated females were then examined for fluorescence to determine the mating partner. The spermathecae containing RhB+ seminal fluid was examined under the florescence microscope equipped with an RFP filter. The spermathecae for females that had mated with an RHB+ (treated) male fluoresced bright orange; those that mated with an unmarked (RhB-) wild type male had a spermathecae which did not fluoresce. The data from the control and two treatment groups were statistically analyzed with the Chi-square test.

## RESULTS AND DISCUSSION

### 1. High levels of mortality are induced in A. stephensi larvae following consumption of Sh.463–56.10R yeast larvicide

**Figure 1:**
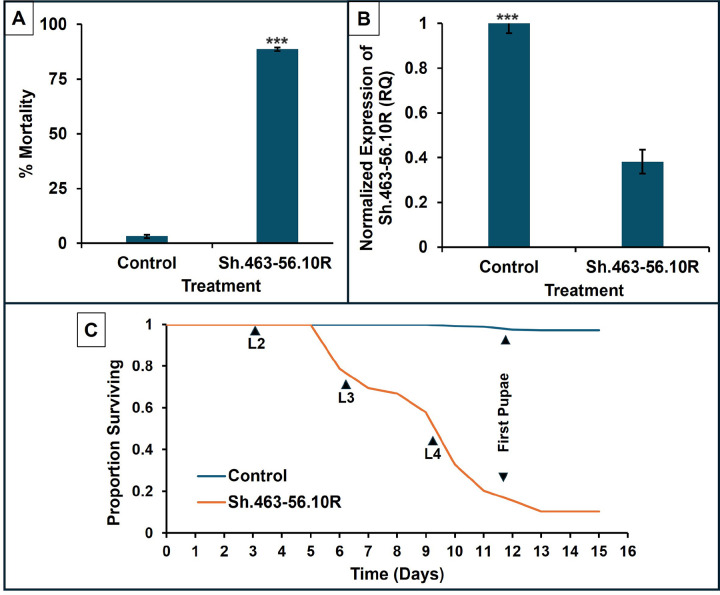
Oral consumption of Sh.463–56.10R yeast induces A. stephensi larval death. *A. stephensi* larval consumption of inactivated dried Sh.463–56.10R yeast induced significant mortality in lab assays. (**A**) Larvicide trials with data represented as mean percentage mortality; error bars represent standard error of the mean (SEM), and *** = *P*<0.001 (Mann-Whitney U test) in comparison to control yeast-treated larvae; (**B**) Silencing of the *Sh* gene target following larval consumption of Sh.463–56.10R yeast. Data represent mean relative quantity (RQ); error bars represent SEM, and *** = *P*<0.001 (Student’s t-test) in comparison to control yeast-treated larvae. (**C**) Survival curves for 1st instar larvae treated with Sh.463–56.10R yeast or control yeast. Consumption of the yeast larvicide induced larval mortality in the third and fourth instars (L3 and L4), days 6–13; compared to larvae fed with control yeast that survived and pupariated). Comparison of the survival curves with the log-rank (Mantel–Cox) test revealed significant differences in survival times between the treatment and control (*P* <0.001).

Our previous high-throughput screens identified hundreds of IRPs, a number of which have target sites that are conserved in many species of disease vector mosquitoes, but not in humans or other non-target organisms, and which kill both larvae and adult mosquitoes [27], [28], [49–51]. Sh.463, one of these IRPs, is an shRNA that can be delivered to mosquitoes in *S. cerevisiae* that are cultured to enable RNA propagation, and then heat-killed, dried, and fed to larvae. Laboratory trials have demonstrated that the yeast kills mosquito larvae of multiple species [27, 28], [50–52], yet did not harm non-target organisms, suggesting that the insecticides could enable eco-friendly *A. stephensi* control. Laboratory trials were therefore conducted on *A. stephensi* larvae using the Sh.463–56.10R[30] yeast strain. As predicted, these laboratory larvicidal assays demonstrated that consumption of the heat-inactivated Sh.463–56.10R yeast effectively killed *A. stephensi* larvae (Fig. 1a).

When treated with the yeast beginning in the first instar, *A. stephensi* larval death occurred during the third or fourth instar stages (Fig. 1c), resulting in mortality rates of 88.2 ± 1% in the treated larvae (compared to control with 3.1 ± 3.4%, *P* < 0.001; Mann-Whitney U t-test). Furthermore, this larval lethality was achieved at dosages that were half (20 mg per 20 larvae) of those used in assays completed with the original first-generation Sh.463 laboratory yeast strain (40 mg per 20 larvae; [28]), which is consistent with recent findings in *Aedes* and *Culex* larvae [30]. That is because the Sh.463–56.10R strain, which was created using Cas-Clover and Super PiggyBac (sPB) transposase/transposon engineering systems, has multiple Sh.463 shRNA expression cassettes that were integrated into the yeast genome [30], enabling increased expression levels of shRNA in each yeast cell, permitting the use of smaller amounts of yeast, which is anticipated to cut the costs for use by mosquito control programs. Moreover, unlike the laboratory Sh.463 yeast strain, the production of Sh.463–56.10R is readily scaled, which would prove invaluable if large-scale field trials and the eventual launching of yeast larvicide distribution were to be pursued [30].

A previous study[39] demonstrated that death of *A. aegypti* mosquito larvae correlated with silencing of the *Sh* gene, which coincided with significant neural defects. It was therefore hypothesized that silencing of the *Sh* gene in *A. stephensi* larvae was responsible for mosquito larval mortality. RT-qPCR assays confirmed 62% silencing of *Sh* gene expression in the *Sh*.463–56.10R yeast-treated larvae (*P* < 0.001; Student’s t-test; (Fig. 1b), indicating that the mode of action for this insecticide in *A. stephensi* larvae is through silencing of the *Sh* gene.

*A. stephensi* has adapted to breeding in a wide variety of man-made containers including water storage tanks, discarded tires, ditches and canals[21] indicating that larviciding, a less commonly used *Anopheles* mosquito control method in Africa, could prove effective for *A. stephensi* control. The WHO, which recommends that larviciding be considered for malaria control in areas where breeding sites are few, fixed, and findable, suggested that larviciding may be a leading method for malaria vector control in urban areas [53]. In 1994, Kumar et al[54] reported decreased *A. stephensi* densities and malaria incidence following *Bacillus sphaericus* larvicide treatment campaigns in India. *B. thuringiensis israelensis* (Bti) and temephos have also been used to target *A. stephensi* larvae [55]. Thus, it is anticipated that Sh.463 larvicides, which are also species-specific, could prove useful in *A. stephensi* larviciding programs. Additionally, RNAi yeast larvicide has been prepared in large slow-release briquette formulations[30] that have demonstrated five months residual activity in semi-field trials conducted on *A. aegypti* larvae in large water storage containers located on a rooftop laboratory in Trinidad [56]. Such formulations might prove useful for treatment of *A. stephensi*, which also inhabit large water storage containers. This could be evaluated in future field trials.

### 2. Consumption of Sh.463–56.10R yeast ATSB induced high levels of adult mortality:

2. Consumption of Sh.463–56.10R yeast ATSB induced high levels of adult *A. stephensi* mortality:

ATSBs, which capitalize on the natural sugar feeding behavior of adult mosquitoes that can be lured to feed on sugar bait that has been laced with an insecticide [57], have proven to be effective in the delivery of insecticides to target adult *Aedes* [58–62], *Culex* [63–65], and *Anopheles*[61] [66],[67] mosquitoes. Despite the potential utility of this intervention, challenges such as optimization of the delivery methods, insecticide resistance, and potential non-target effects threaten the prospective adoption and long-term use of ATSBs [57], [68]. Moreover, the attractiveness of ATSBs to adult mosquitoes compared to available plant sugar sources remains critical. Mosquito-specific Sh.463–56.10R yeast can be delivered in sachet bait stations. The yeast has been shown to increase mosquito attraction to the bait stations that have been successfully used in semi-field trials on adult *Aedes* and *Culex* mosquitoes [69]. In addition to deployment of Sh.463–56.10R yeast as a larvicide, the yeast could therefore also potentially prove beneficial to *A. stephensi* adult control programs, but the yeast has not yet been evaluated in this mosquito species.

**Figure 2:**
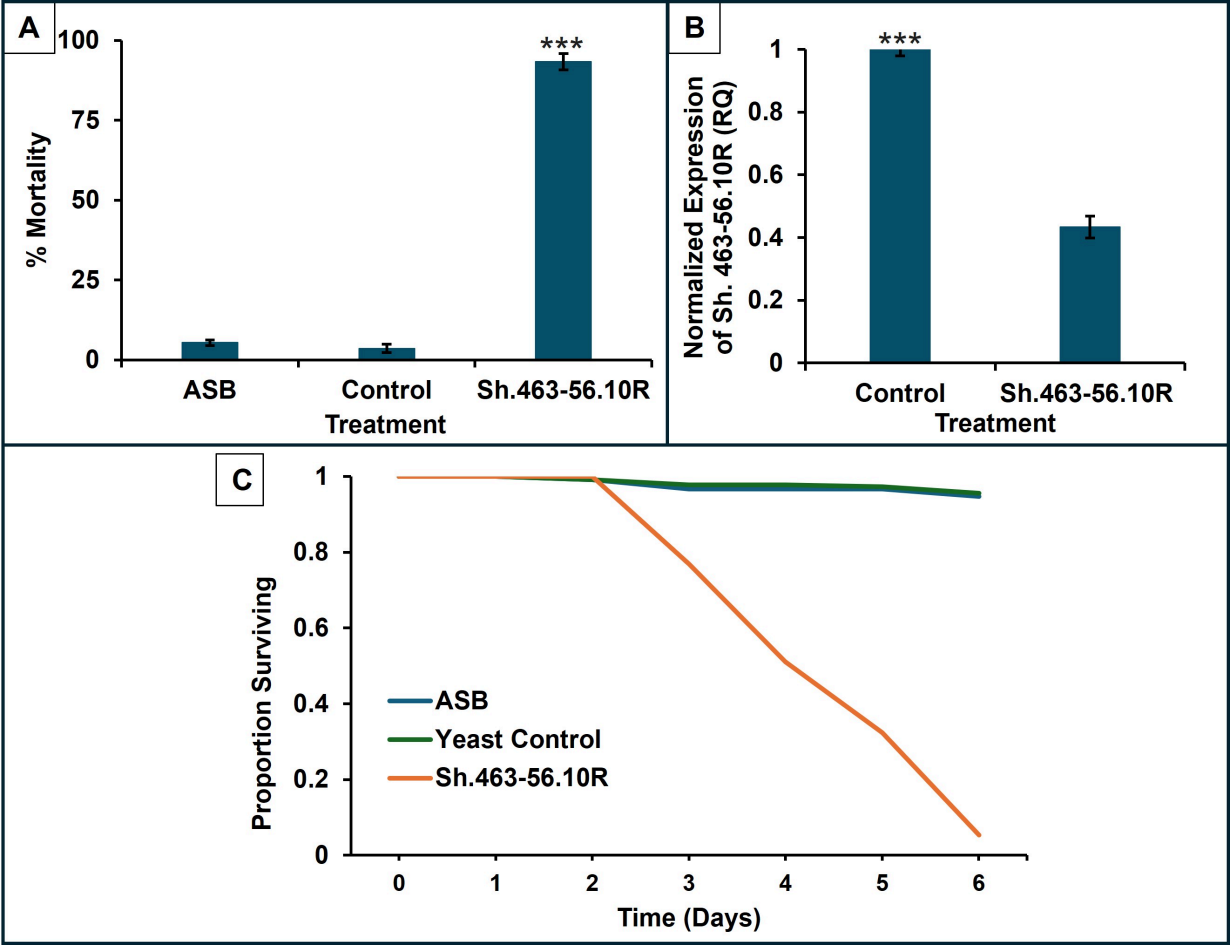
Consumption of Sh.463–56.10R yeast as an ATSB induces high levels of adult A. stephensi deaths Oral consumption of inactivated dried Sh.463–56.10R yeast as an ATSB by adult *A. stephensi* induced significant mortality in lab assays. (**A**) Adulticide trials; data are represented as mean percentage mortality; error bars represent SEM, and *** = *P*<0.001 (Kruskal-Wallis test) in comparison to both Westham sugar and control yeast-treated adults. (**B**) Silencing of the *Sh* gene following consumption of Sh.463–56.10R yeast ATSB by adult *A. stephensi* after 96h. Data are represented as mean relative quantity (RQ); error bars represent SEM, and *** = *P*<0.001 (Student’s t-test) in comparison to control yeast-ATSB treated adults. (**C**) Survival curves for adult *A. stephensi* treated with Sh.463–56.10R yeast-ATSB, Westham bait (ASB) or yeast control-ATSB. Consumption of Sh.463–56.10R yeast-ATSB induced adult mortality on days 3–6; compared to adult *A. stephensi* fed with Westham ASB and control yeast-ATSB, that survived and pupariated. Comparisons of the survival curves with the log-rank (Mantel–Cox) test revealed significant differences in survival times between the treatment and control groups (*P*<0.001).

The Sh.463–56.10R yeast was suspended in Westham sugar bait and fed to adult *A. stephensi* females. Sh.463–56.10R yeast resulted in significant adult mortality with respect to sugar control (Westham bait alone), and control yeast (prepared with Westham bait) treatments, inducing mortality rates of 93.2 ± 1.7% in the treatment compared to 5.3 ± 3% in Westham sugar and 3.6 ± 4% in control yeast bait stations (Fig. 2a; *P* < 0.001 vs. both Westham sugar and yeast control, the mortality of which were not significantly different from each other). Similar observations were made in previous studies involving adult *Aedes* and *Culex* mosquitoes[30] [69]. As illustrated in the survival curve Fig. 2c, adult *A. stephensi* treated with Sh.463–56.10R-ATSB died beginning on day two until day six, when the trial was concluded. Survival curve comparisons with the log-rank (Mantel–Cox) test revealed significant differences in survival times between Sh.463–56.10R yeast ATSB vs sugar and yeast control treatments (*P* < 0.001).

Silencing of the *Shaker* gene was observed in adults that had been treated with Sh.463–56.10R yeast ATSB (57% reduction of the *Shaker* gene transcript, *P* < 0.001; Student’s t-test; (Fig. 2b). *Shaker* gene silencing has resulted in neural defects, including a shaking phenotype that precedes death in *A. aegypti* [28]. In addition to loss of flying, Sh.463–56.10R yeast also resulted in a shaking phenotype similar to those observed in *A. aegypti* [28], suggesting that silencing of *Sh* results in neural defects that contribute to the death of *A. stephensi*.

**Figure 3:**
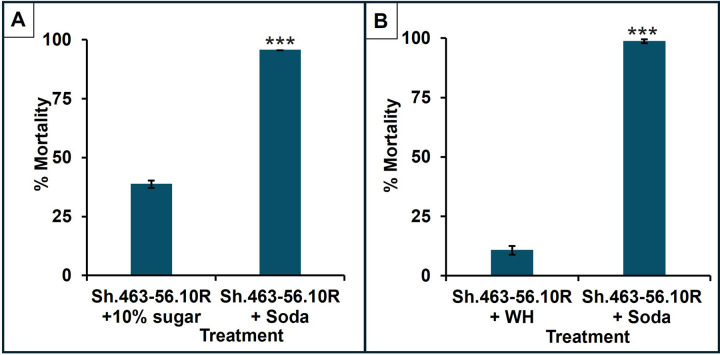
Soda is the preferred sugar bait in adult A. stephensi ASB choice assays. The attractiveness of 10% sugar, Westham bait, and soda for the delivery of Sh.463–56.10R yeast as an ATSB to adult *A. stephensi* is demonstrated as a function of mortality. Compared to the Sh.463–56.10R-Westham bait or Sh.463–56.10R-10% sugar-yeast formulations, significantly higher levels of adult female mortality were observed when Sh.463–56.10R insecticidal yeast was delivered using soda as a sugar bait (Fig. 3a, 10% sugar-Sh.463–56.10R ATSB vs. soda-Sh.463–56.10R ATSB; Fig. 3b, Westham sugar-Sh.463–56.10R ATSB vs soda-Sh.463–56.10R ATSB; error bars represent SEM, and *P* = 0.002, Mann-Whitney U test). ASB: attractive sugar bait; ATSB: attractive targeted sugar bait.

If RNAi-ATSBs are to be useful for control of *A. stephensi*, it is critical that the yeast-bait stations successfully attract these mosquitoes away from natural sugar sources. It was hypothesized that Coca-cola^™^ (hereafter referred to as soda), which has proven to be an excellent sugar bait for *Aedes japonicus*[70] and *Drosophila suzukii* [39], might prove to be a highly attractive sugar bait for *A. stephensi*. The attractiveness of 10% sucrose solution (commonly used in laboratory sugar feeding assays), Westham bait, and soda for the delivery of Sh.463–56.10R yeast pesticide to adult *A. stephensi* was examined as a function of mortality. Compared to the yeast-Westham bait or 10% sucrose-yeast formulation, significantly higher levels of adult female mortality were observed when the yeast insecticide was delivered using soda as a sugar bait (Fig. 3a, soda vs. 10% sugar, 96%± 4%, vs. 39%±4%, *P* = 0.002; Fig. 3b, soda vs. Westham sugar, 99%±1% vs. 11%±2%, *P* = 0.002). The higher mortality rates in treatment cages with soda-yeast ATSB vs. other sugar baits demonstrate the adult females’ higher preference for soda as a sugar source. Similar results observed in *Aedes*, *Culex* and *A. gambiae* adults, which will be discussed elsewhere, suggest that the use of soda for the delivery of RNAi yeast may be beneficial for control of many different disease vector mosquitoes.

The results of these trials suggest that Sh.463–56.10R yeast could be deployed in bait stations to kill *A. stephensi* adult mosquitoes, and that soda may effectively compete against natural sugar sources, which must still be assessed in the field. Plant sugar feeding is a dietary requirement for both male and female adult mosquitoes [71], and plant sugar feeding in the home environment, both indoors and outdoors, has been described in malaria mosquitoes [72]. Moreover, *A. stephensi* obtains blood meals from both humans and animals and exhibits more outdoor feeding[73], suggesting that the use of RNAi yeast ATSBs could prove effective for mosquito control in the home environment.

### 3. Female-specific yeast larvicides facilitate male mosquito sorting in

3. Female-specific yeast larvicides facilitate male mosquito sorting in *A. stephensi*

**Figure 4:**
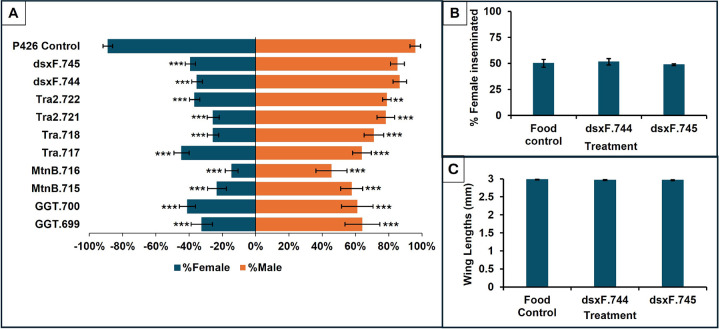
Larvicidal screen of A. stephensi putative female-specific larval lethal genes for male mosquito sorting Larval consumption of the *GGT*.699, *GGT*.700, *MtnB*.715, *MtnB*.716, *Tra*.717, *Tra*.718, *Tra2*.721, and *Tra2*.722 yeast larvicides resulted in significant female and male deaths with respect to control yeast (Fig. 4a, Chi- square, ***= *P* < 0.001). *A. stephensi* larval treatment with *dsx*F.744 and *dsx*F.745 yeast larvicides targeting the female-specific *dsx* gene resulted in the highest male: female ratios, with 4 female: 1 male ratios in the surviving offspring (Fig. 4a, Chi- square, ***= *P* < 0.001). Larval consumption of *dsx*F.744 and *dsx*F.745 yeast larvicides did not significantly impact *A. stephensi* males’ mating competitiveness (Fig. 4b, and *P* > 0.05 (Chi- square for each of three experiments in which males of the indicated treatments competed for mating with wild type females), or wing lengths (Fig. 4c, *P* > 0.05, with respect to food control. Error bars represent SEM.

In addition to insecticides, population-based control strategies such as SIT or release of insects carrying a dominant lethal (RIDL) [74], [75], could prove valuable in the fight against *A. stephensi*. However, these strategies often rely on the release of mating-competitive adult males, and efficient, effective, and globally deployable methods for scaled production of males can be a barrier to the development of such programs [78–81]. Previous studies demonstrated that yeast RNAi-mediated silencing of genes such as *MtnB* and *GGT* in the M/m sex-determining locus region of *A. aegypti* during larval development resulted in death of female larvae. Moreover, silencing the orthologs of these genes in *A. albopictus* and *Culex spp. larvae* killed females [31],[32] leading to the hypothesis that silencing of the *A. stephensi MtnB* and *GGT* orthologs would kill female larvae. To test this, yeast larvicides corresponding to the *A. stephensi MtnB* and *GGT* genes were prepared and fed to *A. stephensi* larvae. Larval consumption of *Mtn*B.715, and *MtnB*.716 yeasts (targeting the *MtnB* gene), *GGT*-A.699, and *GGT*-B.700 (targeting the *GGT* gene) yeasts resulted in significant female deaths with respect to control yeast treatment (Fig. 4a, Chi- square, ***= *P* < 0.001). These deaths occurred during the third instar stage. However, targeting the *MtnB* or *GGT* genes also resulted in significant death of males (Fig. 4a, Chi- square, ***= *P* < 0.001), indicating that these larvicides were not good choices for use as sex-separation tools. This was somewhat surprising given that silencing of these genes, which are located adjacent to the *A. aegypti* sex-determining M/m locus, results in female-specific larval lethality in *Aedes, Culex*, and *A. gambiae* mosquitoes and production of fit adult males [31, 32]. It is possible that these genes are required in *A. stephensi* male larvae, or perhaps the larvicides, which were designed to be as gene-specific as possible, have unintended off-targeting effects on other necessary loci.

The *doublesex (dsx)* gene, a key regulator of sex-specific development[80][81] in many insects including *A. stephensi*, has both male (*dsx*M) and female-specific (*dsx*F) transcripts (Gene Bank, *dsx*M: KP257287.1; *dsx*F: KP257286.1). In *Drosophila*, the differential splicing of the female-specific *dsxF* transcript is regulated by *transformer* (Tra) and *transformer*2 (Tra2) [82], [83]. Yeast larvicides corresponding to the female *A. stephensi dsx* transcript, *dsx*F.744 and *dsx*F.745 as well as larvicides corresponding to the *tra* (*Tra*.717 and *Tra*.718) and *tra2* genes (*Tra2*.721, *Tra2*.722) were generated and assessed through larval feedings in the lab. Although silencing of *dsxF* resulted in female-specific lethality, silencing of *tra* and *tra2* resulted in death of both *A. stephensi* males and females (Fig. 4a, Chi- square, *** = *P* < 0.001). It is possible that off-targeting by the Tra.717, Tra.718, *Tra2*.721, and *Tra2*.722 larvicides leads to silencing of genes other than *Tra* and *Tra2*. Alternatively, perhaps *Tra* and *Tra2* have taken on additional critical roles in *A. stephensi* males.

Of the putative female-specific larvicides tested, the *dsx*F.744 and *dsx*F.745 larvicides resulted in the highest male: female ratios, with 4 female: 1 male ratios in the surviving offspring. Moreover, no significant male death was detected, and no significant impact on male survival (Fig. 4a, P < 0.001) or fitness, as assessed by the estimation of wing lengths (Fig. 4c, P > 0.05, *dsx*F *vs*. food control treated males) and competitive mating (Fig. 4b, P > 0.05, *dsx*F *vs*. food control treated males), were found. Similar results were obtained when Taracena et al.[84] used a bacterial system that targeted the female transcript of *A. gambiae*. Additionally, Whyard et al.[85] successfully used RNAi to silence the female-specific *dsx* transcript in *A. aegypti* larvae through oral feeding assays conducted using an *E. coli* dsRNA expression system, which resulted in female-specific deaths following larval consumption.

The inclusion of these female-specific yeast larvicides in *A. stephensi* control programs that rely on large-scale male releases could be valuable. As demonstrated in *Culex* mosquitoes [35], scaled production of female-specific yeast larvicides can be achieved using industrial-scale robust yeast strains that may enhance the efficacy, efficiency, and cost-effectiveness of male sorting technology. Although the female-specific larvicide clearly cannot serve as a standalone technique for sex separation, it could likely be used in conjunction with other sex separation techniques to increase productivity and speed of the sorting process. Moreover, given that it acts during the third instar, it could help reduce costs associated with mass rearing mosquitoes. In this manner, the use of yeast RNAi female-specific larvicides targeting *dsxF* in *A. stephensi* could promote the design of globally deployable strategies to improve male sex separation, which is often required for population-based mosquito control strategies [74].

## CONCLUSIONS

Here we demonstrated that RNAi yeast can be used as an effective larvicide, adulticide, or male sex separator in *A. stephensi*. Larviciding is a key method for controlling *A. stephensi* larvae, and the generation of a new class of eco-friendly RNAi yeast larvicides, which could be used in rotations with existing larvicides, is likely to benefit long-term larviciding campaigns. Moreover, it could be used in conjunction with RNAi yeast ATSBs for integrated mosquito control. Given the outcomes of the recent field trials conducted with the Westham bait station in Africa, which indicated that the ATSB stations did not reduce the incidence of malaria [86], [87], the increased attractiveness of the yeast-soda combination (in ATSB bait stations) could prove to be useful in integrated *A. stephensi* control programs, but this will need to be further assessed in future field trials, in which the residual activity of the yeast sugar baits can be further evaluated. Finally, the addition of a male sex separator in mass larval rearing diets is likely to facilitate SIT campaigns. Weng et al.[88] have elegantly pursued generation of a separator strain that enables precise male selection from the first instar larval stages. However, the yeast method is more portable and doesn’t require the use or alteration of transgenic strains. In summary, we have succeeded in the generation of new RNAi yeast strains for the control of *A. stephensi* mosquitoes. Our studies demonstrated that the RNAi yeast can be used for successful larviciding, adulticiding, and sex-separation in support of mosquito control programs.

## Supplementary Material

Supplementary Files

This is a list of supplementary files associated with this preprint. Click to download.

- FigureS1.jpg

## Data Availability

All data generated or analyzed during this study are included in this published article [and its supplementary information files].

## Abbreviations

ANOVA: Analysis of Variance
ASB: Attractive Sugar Bait
ATSB: Attractive Targeted Sugar Bait
cDNA: complementary DNA
DNA: Deoxyribonucleic acid
dsRNA: double stranded RNA
*dsx*F: Female-specific doublesex
*dsx*M: Male-specific doublesex
*GGT*: Gamma-glutamyltransferase
IRP: Interference RNA pesticide
LC_50_: Lethal Concentration which kills 50% of test the animals
LC_90_: Lethal Concentration which kills 90% of test the animals
MR: Mass Rearing
*MtnB*: Metallothionein B
PCR: Polymerase chain reaction
qPCR: Quantitative PCR
RFP: Red Fluorescent Protein
RhB: Rhodamine B
RIDL: Release of Insects carrying a Dominant Lethal
RNA: Ribonucleic acid
RNAi: RNA interference
*rps7*: ribosomal protein 7
RT-qPCR: Reverse Transcription quantitative PCR
SEM: Standard Error of the Mean
*Sh*: Shaker
shRNA: short hairpin RNA
siRNA: small interfering RNA
SIT: Sterile Insect Technique
SSA: Sub-Saharan Africa
Tra: Transformer
Tra2: Transformer 2
WH: Westham
WHO: World Health Organization
